# Safety and Efficacy of an Atraumatic Uterine Cervical Traction Device: A Pilot Study

**DOI:** 10.3389/fmed.2021.742182

**Published:** 2021-12-23

**Authors:** Hélène Legardeur, Gessica Masiello-Fonjallaz, Martine Jacot-Guillarmod, Patrice Mathevet

**Affiliations:** Gynaecology, Woman Mother Child Department of the Lausanne University Hospital (CHUV), Lausanne, Switzerland

**Keywords:** intrauterine contraceptive device, pain, cervix, atraumatic tenaculum, bleeding

## Abstract

**Introduction:** Alignment of the uterine cervix with the vaginal canal is often required during insertion of an intrauterine contraceptive device (IUD). Currently available instruments are traumatic tenacula, which can cause pain and bleeding and represent an obstacle for certain patients to pursue their medical follow-up. A novel investigational cervical vacuum tenaculum, enables atraumatic traction of the cervix using a semi-circular suction pad, designed to conform to the anatomical shape of the external cervical os. Suction is generated by manually pulling out a sliding tube in a vacuum chamber.

**Methods:** We performed a single arm non-comparative pilot study to assess the safety and efficacy of the cervical vacuum tenaculum in 13 women receiving an IUD. Data on procedural efficacy, safety, patient-reported pain scores at specific time points during IUD insertion procedure and patient satisfaction were collected prospectively.

**Results:** Insertion of IUD was successful with use of the study device in 7 of the 13 enrolled patients (54%). No bleeding or only limited ecchymosis were caused by the device. No adverse events were reported. Participants reported very little pain (mean Visual Analog Scale <10) when applying the device. Participants who achieved IUD insertion with the device reported strong overall satisfaction with the procedure.

**Conclusions:** The suction-based atraumatic tenaculum can be used to manipulate the cervix during IUD insertion with satisfactory efficacy and safety. The results of this pilot study support further studies of this device in larger populations comparing with standard single-tooth tenaculum.

**Clinical Trial Registration:** ClinicalTrials.gov, identifier: NCT 04441333.

## Introduction

Intrauterine contraceptive devices (IUDs) are highly effective long-acting, well-tolerated and safe reversible contraceptive methods. IUD placement requires access to the uterus through the cervical canal, which often involves grasping the cervix and applying traction to align the uterus, cervical opening and vaginal canal. The use of a single-tooth cervical (Pozzi) tenaculum, a two-pronged instrument that penetrates opposing points into the cervical stroma, is commonly used to hold and manipulate the cervix. However, engaging the tenaculum on the cervix is associated with pain both during the procedure and post-procedurally (1, 2).

Uterine sounding and IUD insertion are further painful steps in the procedure (3). Procedural anxiety may also be associated with higher pain scores at the time of tenaculum placement (3, 4). In consequence, fear of pain during IUD insertion is a limitation to their use (5, 6).

There is no consensus on effective analgesics to reduce pain (1), nor on the use of different tenaculum designs (3). Alternatives to the Pozzi tenaculum include the curved Teale vulsellum with multiple small teeth that are not intended to puncture the cervical mucosa to grasp the cervix at the time of IUD insertion, Littlewoods forceps or the Allis forceps. Available data indicate similar pain levels with all these aids (3, 7, 8). Several studies have assessed the use of analgesics, local anesthetic or misoprostol to soften and ripen the cervix, with contradictory results (1, 9–11). Procedural interventions for pain management include different types and designs of tenacula. However, it has not been possible to demonstrate a statistically significant reduction in pain scores during tenaculum placement, regardless of the type used (3).

This pilot study assessed the efficacy and safety of an investigational soft-suction device for atraumatic stabilization of the cervix during the insertion of an IUD.

## Methods

This was an interventional open-label pilot study, with the aim to assess the efficacy and safety of an atraumatic device during IUD insertion. Included were patients presenting for insertion of a 52 mg levonorgestrel IUD at the gynecology outpatient clinic of the Lausanne University Hospital. Eligible participants were aged 18 years or older. Subjects were excluded if on anticoagulant medication or pregnant, or if presenting with a cervix diameter <26 mm, cervical abnormalities including carcinoma, cervical dysplasia, previous cervical operation or severe vaginal bleeding. If upon examination no instrument for cervical traction was found to be required for IUD insertion, patients were also excluded. Excessive alcohol, drug, benzodiazepine or anesthetic use prior to the procedure was not allowed. All participants provided written, informed consent.

### Study Device

**Figure 1:**
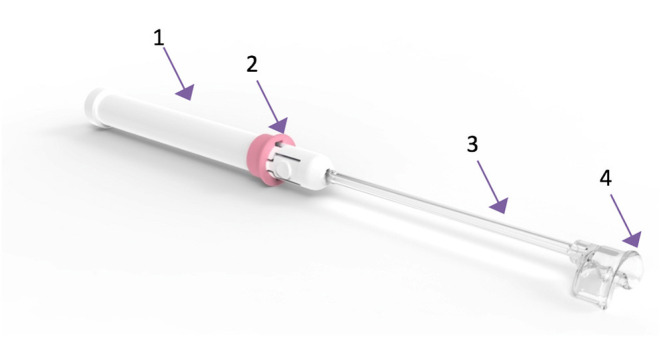
The suction-based atraumatic tenaculum used in the study. 1. Main body; 2. Slider ring to apply and release suction; 3. Sliding tube to generate vacuum; 4. Semi-circular suction pad for affixation to the cervix.

The cervical vacuum tenaculum (Aspivix SA, Renens, Switzerland) is an investigational, atraumatic device which uses suction force to hold and manipulate the cervix during IUD insertion. The sterile, single-use device (Figure 1) features a semi-circular suction pad, designed to follow the external cervical os anatomy. A vacuum is created within the main body of the device by pulling out the sliding tube. The device is then lightly affixed to the external cervical os. Suction is applied by pushing the slider ring. The device can be reloaded in case of vacuum loss. Tissue is released by releasing the vacuum using the sliding tube.

### Procedure

Three physicians performed the study procedures. All operators were trained on a silicone cervix mock-up to model device functionality, especially how to generate the vacuum and the interaction with the cervix. The insertion of the IUD was performed according to the hospital's standard guidelines. After speculum exposure, the cervix was cleaned with appropriate antiseptic. The operator inspected cervix anatomy to rule out any contraindications and confirm that cervical traction would be required for IUD insertion. The surface of the cervix was dried with a dry and sterile swab. The operator placed the suction pad of the study device in contact with the cervical outer surface and activated the suction by pushing the slider (Supplementary Figure). Operators were instructed to allow at least 3 s between vacuum deployment and cervix manipulation. The subsequent IUD insertion procedure continued as per standard hospital procedures. In case of premature release of the device, vacuum was re-created and the device was re-applied. If a second loss of vacuum occurred, the device was replaced. During the entire procedure, no prophylactic cervical anesthesia was used.

### Data Collection and Outcomes

At study entry, data were collected on demographics, obstetric and gynecological history and any use of pain medication in the 24 h prior to the procedure.

The primary objective was to assess the safety and efficacy of the suction-based atraumatic tenaculum. Patient satisfaction was evaluated using a five-point Likert questionnaire (Appendix) after the IUD insertion procedure.

Secondary objectives were to assess patient reported pain during the IUD insertion, as well as overall patient satisfaction and treating physicians' experiences with the novel device. Pain scores were assessed using a 100-point Visual Analog Scale (VAS) at 7 stages of IUD insertion: before the procedure, during speculum insertion, during suction application, during application of cervical traction, during IUD insertion through the cervical canal, during release and 5 min after the end of the procedure.

Safety was evaluated by adverse events, noted by the treating physician who recorded bleeding or ecchymosis and the relation to the study intervention. Between 3 and 5 days post-insertion, the practitioner called study subjects to inquire into use of co-medications, post-procedural bleeding or any other safety concern arising after the procedure.

### Statistical Methods

Data are presented descriptively as mean, standard deviation, median, range and interquartile range for continuous data, or number and percentages for discrete data. For this pilot study a sample size of 10 participants was calculated based on 80% power to observe at least one device failure or adverse event if these were to occur in at least 15% of participants.

### Ethical Approval

The study was carried out in accordance with the protocol and with the principles laid out in the contemporaneous version of the Declaration of Helsinki; the European Directive on medical devices 93/42/EEC and ISO Norms 14155 and 14971; as well as in compliance with Swiss Law and the requirements of the Swiss regulatory authority. The trial is registered on ClinicalTrials.gov (NCT0444128) and on the SNCTP (Swiss National Clinical Trials Portal).

## Results

### Study Population

From July to November 2020, 13 patients were enrolled. Mean age was 36.3 ± 10.5 years (range: 21–57 years), mean height 164.1 ± 5.5 cm (150–173 cm) and average weight 58.0 ± 6.7 kg (48–70 kg). Nine patients were Caucasian. Three patients had no history of pregnancy. No patients were excluded after inspecting the anatomy of the cervix and all patients needed cervical traction to facilitate IUD insertion. However, for one participant a contraindication (Nabothian cyst) was identified after inclusion; in this subject IUD insertion was successful using standard tools.

**Figure 2:**
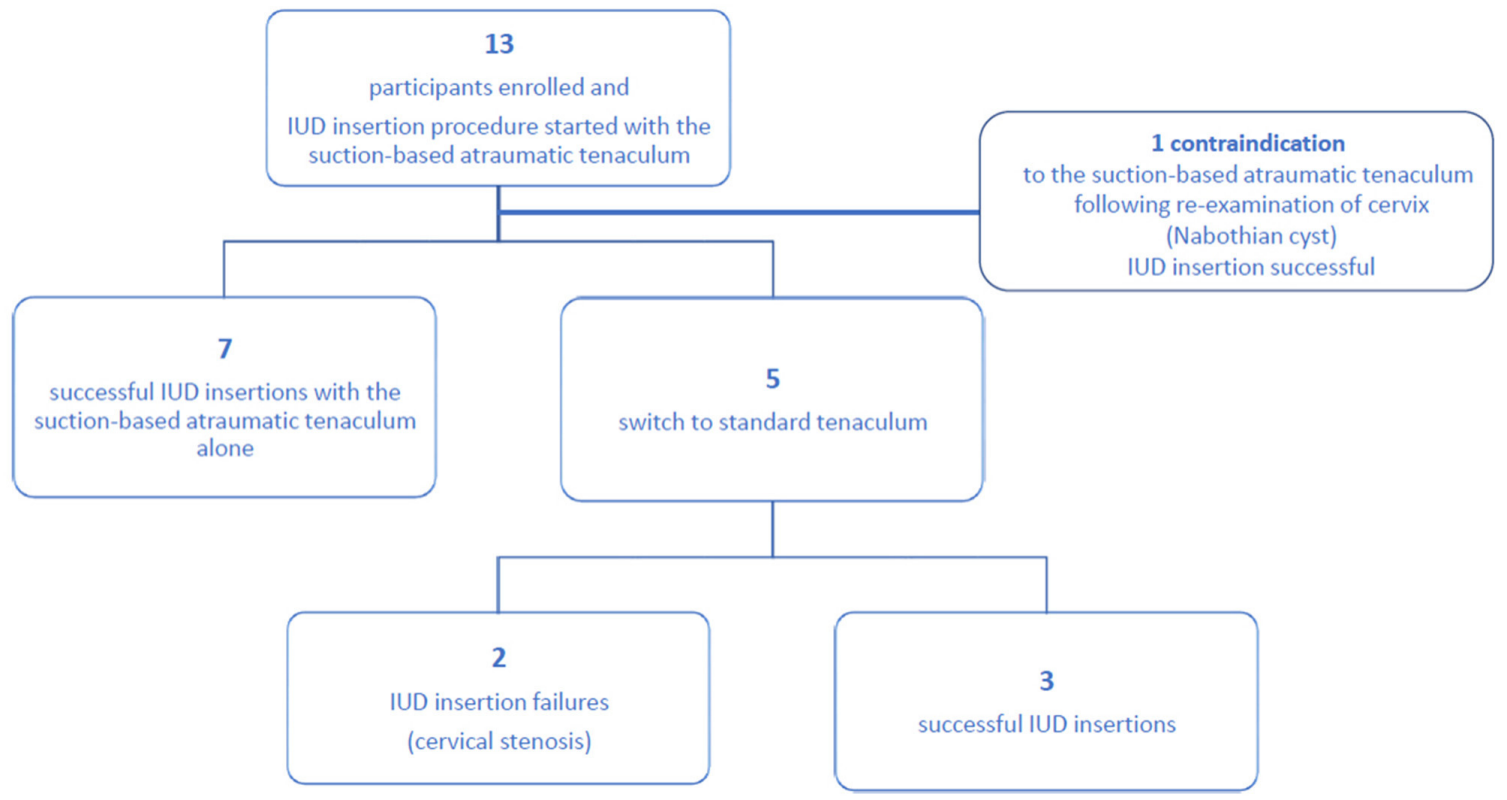
Study flow chart.

The flow of patients is shown in Figure 2. Two practitioners performed 12 of the 13 interventions. The IUD insertion was successful in 11 subjects (85%); seven with aid of the suction-based atraumatic tenaculum and 4 (including the subject contraindicated for the study device) after switching to standard single-tooth tenaculum. In 9 out of 13 cases, the practitioner reported spontaneous, often recurrent releases of the device from the grasped tissue. There were two releases in two successful procedures, and one release in two successful procedures, respectively. The two unsuccessful procedures, after attempts with suction-based and standard tenaculums, were due to cervical stenosis.

### Patient-Reported Pain

Complete participant-reported data were only available for subjects who experienced a successful IUD using the suction-based tenaculum (Table 1). Very low pain scores were reported before the procedure (average VAS score 0), during speculum insertion (mean VAS score 1.5 ± 4.3), while the suction-based atraumatic tenaculum was applied (mean VAS score 7.7 ± 10.5) and during the application of cervical traction (mean VAS score 12.2 ± 11.3). No patient required adjuvant pain medication. The 7 successfully treated participants strongly agreed that they were overall very satisfied with the procedure. The 6 subjects who were switched to standard tenaculum device were not required to provide satisfaction scores.

**Table 1:** Participant-reported VAS pain scores at different stages of the procedure.

	**Pre-procedure**	**Speculum**	**Suction**	**Cervical traction**	**IUD insertion**	**Device release**	**Post-speculum**
		**placement**	**application**	**application**			**removal**
*n* ^*^	13	13	13	12	8	7	8
Mean VAS ± SD	0.0 ± 0.0	1.5 ± 4.3	7.7 ± 10.5	12.2 ± 11.3	27.8 ± 22.7	11.4 ± 20.4	9.8 ± 16.9
Median VAS [IQR]	0 [0–0]	0 [0–0]	4 [0–10]	9 [2–23.5]	35 [3.5–47.5]	0 [0–30]	2 [0–10]

* *Complete data were only available for participants who experienced a successful procedure*.

During the first 5 days following the intervention, 2 of the 13 participants experienced moderate pain; both had used a standard tenaculum. No patients reported abnormal bleeding after the procedure. No study device-related adverse events were reported.

### Operator Feedback

The practitioners' opinions on the device depended on whether the IUD insertion could be accomplished successfully with the study device alone or whether the operator switched to standard tenaculum (Table 2). After the seven successful IUD insertions using the study device, all practitioners agreed or strongly agreed that the device facilitated pulling and alignment. Most (6/7) agreed or strongly agreed that the device provided adequate visibility and that overall handling was satisfactory, and five agreed or strongly agreed that they felt confident with the grasping quality. After the six procedures where a switch to standard tenaculum was necessary, satisfaction scores were substantially lower, although 4/6 operators found overall handling satisfactory and 2/6 felt confident with the grasping quality and/or were satisfied with the visibility. No physician found the device unnecessarily complicated to use. Among other positive characteristics mentioned by operators were the lack of pain, absence of trauma to the cervix and easy maneuverability.

**Table 2:** Satisfaction scores among operators, according to procedural success with the Carevix device.

**Assessment**	**Agree/strongly agree**		**Indifferent**		**Disagree/strongly disagree**	
	**Successful**	**Switched to**	**Successful**	**Switched to**	**Successful**	**Switched to**
	**procedure**	**standard tenaculum**	**procedure**	**standard tenaculum**	**procedure**	**standard tenaculum**
The insertion could be performed as planned	5	0	0	1	2	5
The device provides adequate visibility/access of the cervix during the procedure	6	2	1	2	0	2
I felt confident with the grasping quality of the cervix by suction	5	2	2	1	0	3
The device facilitates pulling and aligning the cervix with the uterine cavity	7	0	0	1	0	5
The overall handling of the device was satisfactory	6	4	1	4	0	2

### Safety

A total of 21 devices were used in the 13 subjects. Two devices were used inappropriately (one inappropriate cleaning attempt; one inappropriate locking of the vacuum mechanism) and three were defective (two cases of inability to maintain vacuum and one device with a part detaching from the handle during vacuum generation before employment). Three devices were used as a replacement after failed attempts to grasp the cervix. No defect was associated with an adverse event or any consequence to the study participants. One minor adverse event occurred, due to malfunction of the IUD inserter. The event was not considered related to the investigational device or procedure, and did not have any further consequences for the participant. Using a second IUD, the procedure could be performed successfully without sequelae. No bleeding was associated with use of the study device and only limited ecchymosis <1 cm, in 5 cases.

## Discussion

In this pilot assessment of the atraumatic cervical vacuum tenaculum involving three operating physicians and 13 subjects, the device could be successfully and safely applied in the majority of subjects, with no need for adjuvant pain medication. The effectiveness of the device was dependent on the appropriate application of vaccuum. Patient satisfaction with the successfully performed procedures was high, with little or no pain and no use of adjuvant pain medication. Operators were overall satisfied with handling and visibility, and appreciated the lack of pain, absence of trauma to the cervix and maneuverability of the device. No cervical bleeding and no trauma occurred during the procedure.

Patient-reported pain scores from successful procedures were favorable, although because pain scores were only provided by successfully treated subjects, we were unable to perform a direct comparison with standard tenaculum procedures. VAS pain scores have recently been reported with the Bioceptive suction cervical retractor (12) at procedural stages similar to our assessments. Median VAS scores from the acceptance testing with the Bioceptive device were higher than with the Aspivix device at all stages of the procedure, and the upper quartiles were more severe. Given the small sizes and different designs of these studies only limited conclusions can be drawn, but the possibility of reduced pain with the Aspivix device seems worthy of further study. Less pain during cervix suction and traction would be a desirable outcome of a novel device, as it might reduce contraction and pain during IUD insertion. The pain scores during insertion in the current study were similar or lower than in other published scores (3, 12) and a direct comparison may be needed to confirm or reject the hypothesis.

The use of the suction-based atraumatic tenaculum is associated with a learning curve. To apply the device successfully, the practitioner needs to push on the cervix to create the vacuum, which is contrary to the usual procedure with a single-tooth tenaculum. There were signs of a learning curve: although procedure times were not formally recorded, the two practitioners performing the majority of the interventions noted that procedure times decreased and device maneuverability increased with experience.

Physical examination of the cervix is important to determine the optimal conditions for use of the suction-based atraumatic device. If the cervix is tilted or cannot be well-exposed with the speculum, the device might not attach firmly and the risk of release might increase. During the current pilot study, all participants were considered eligible for IUD insertion using the study device. Greater familiarity with the device may improve success rates by enabling the identification and focus on the most suitable patients. Also, the operators' experience indicated that a longer interval (up to 10 s) between vacuum deployment and cervix manipulation was associated with lower risk of release. This was not formally studied but a longer time period will be recommended in the updated instruction manual.

The experience reported here also provides guidance to improvements to the study device. The availability of two head sizes might expand the range of anatomies suitable for treatment and increase the efficiency of the procedure. Improvements targeting the spontaneous, often recurrent release of the suction-based tenaculum would be desirable to increase device adhesion and tensile strength. The application of grease and primer in the device-assembly process needs to be standardized to ensure consistent and reliable vacuum generation. Variations in rod length and position of the vacuum release button might be worth considering as well.

As a pilot study, the current work has a number of limitations. The study population was small. The study was non-randomized, without a control group and only successfully treated patients provided pain scores. No selection of patients based on physical examination was performed and the most suitable patients need to be identified. It can be expected that some of the subjects would have been excluded after examination if physicians had benefited from greater experience with the study device. Generalizability is further limited since cervix size <26 mm was an exclusion criterion, as the investigational device was only available in one size at the time of the study. Furthermore, two practitioners performed all except one of the procedures and the generalizability of the physician assessments may be limited.

## Conclusion

This pilot study indicates that the suction-based atraumatic tenaculum is an effective and safe alternative to standard single-tooth tenaculum, with favorable patient-reported pain scores and satisfaction after successful procedures. There is some scope for design improvement of the device and further clinical experience would enable characterization of the most suitable target population. A follow-up study is ongoing, comparing the second-generation device with standard single-tooth tenaculum, taking into account an IUD failure rate of about 15%, as indicated in the pilot study.

## Data Availability Statement

The original contributions presented in the study are included in the article/Supplementary Material, further inquiries can be directed to the corresponding author/s.

## Ethics Statement

The studies involving human participants were reviewed and approved by Swissmedic. The patients/participants provided their written informed consent to participate in this study.

## Author Contributions

HL drafted the manuscript. GM-F, MJ-G, and PM provided constructive input and approved the manuscript. All authors participated in study design, conduct and analysis.

## Funding

The study was sponsored by ASPIVIX SA. The sponsor provided study devices and compensated the University Hospital for each patient included in the study.

## Conflict of Interest

The authors declare that the research was conducted in the absence of any commercial or financial relationships that could be construed as a potential conflict of interest.

## Publisher's Note

All claims expressed in this article are solely those of the authors and do not necessarily represent those of their affiliated organizations, or those of the publisher, the editors and the reviewers. Any product that may be evaluated in this article, or claim that may be made by its manufacturer, is not guaranteed or endorsed by the publisher.
