# Supplementary material for: Safety and Efficacy of an Atraumatic Uterine Cervical Traction Device: A Pilot Study
Source: Front Med (Lausanne). 2021 Dec 23;8:742182. doi: 10.3389/fmed.2021.742182 (PMC8732360; doi:10.3389/fmed.2021.742182)
Supplement: Supplementary file 1 [file Data_Sheet_1.PDF]

ASPIVIX_IUD2019

**N° identification du sujet :** ___ - ____

# ÉVALUATION DE LA DOULEUR

Lorsque l'assistant vous le demande, veuillez s'il vous plaît indiquer votre niveau de douleur par un trait vertical sur l'échelle correspondante ci-dessous.

| | **Échelle de la douleur** |
| --- | --- |
| | Aucune douleur — Douleur maximale imaginable 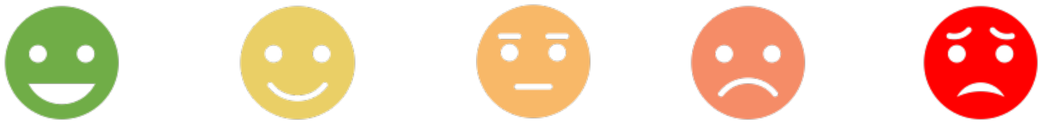 |
| **A** | 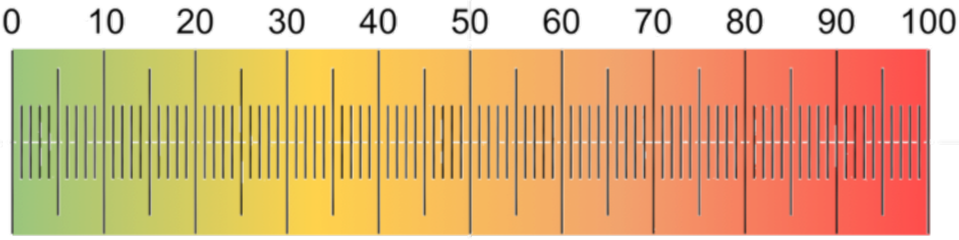  |
| **B** | 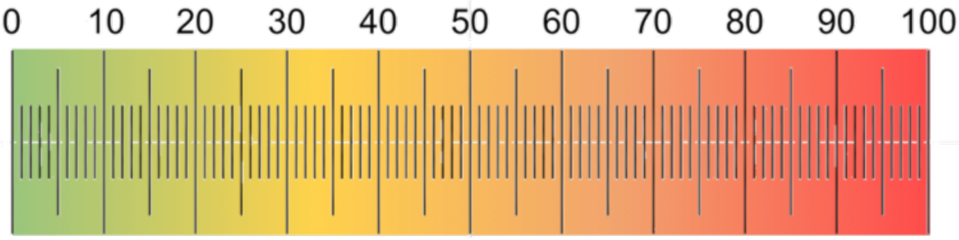  |
| **C** | 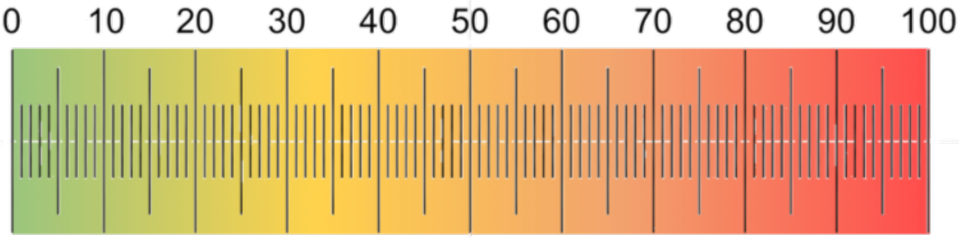  |
| **D** | 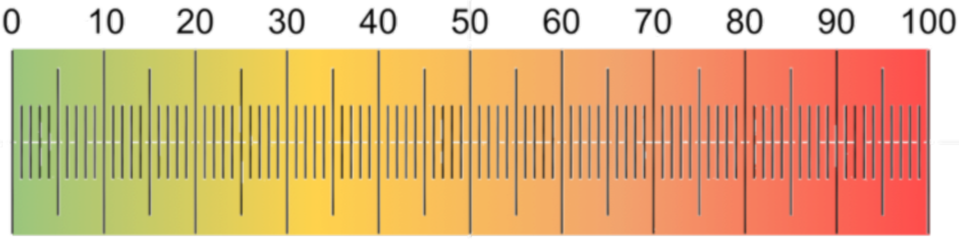  |
| **E** | 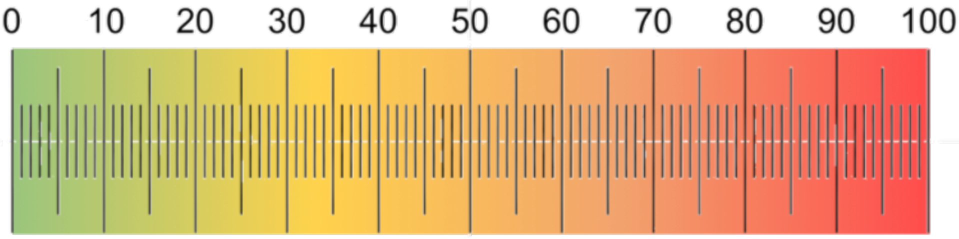  |

**N° identification du sujet :** |_|_|_| - |_|_|_|_|

## Échelle de la douleur

Aucune douleur

Douleur maximale imaginable

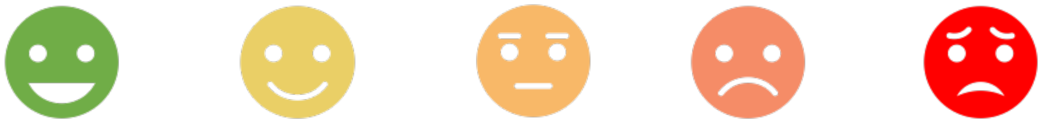

**F**

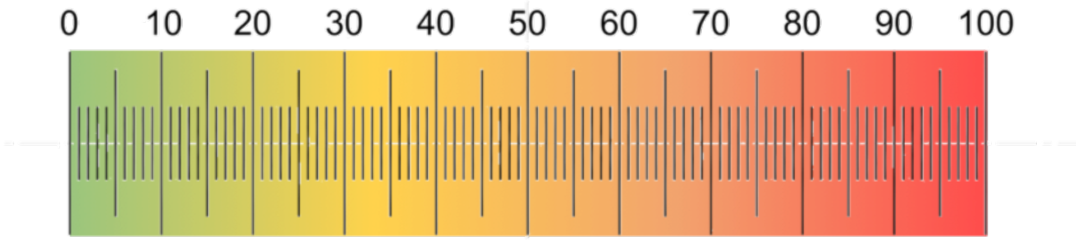


**G**

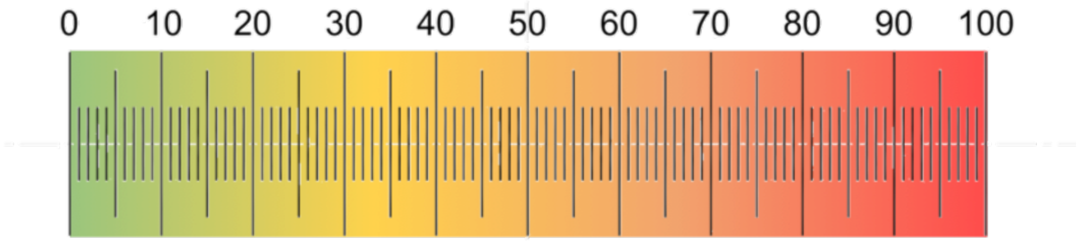


## SATISFACTION

Veuillez s'il vous plaît indiquer votre niveau de satisfaction relatif à la procédure en marquant une croix dans la case correspondante ci-dessous.
Vous pouvez aussi nous faire part ici de toutes remarques ou questions concernant la procédure.

| | Pas du tout d'accord | Pas d'accord | Indifférente | D'accord | Tout à fait d'accord |
|---|---|---|---|---|---|
| De manière générale, je suis très satisfaite de la procédure. | | | | | |

| | |
|---|---|
| Avez-vous des remarques ou questions ? | |
